# A genetic predictive model for precision treatment of diffuse large B-cell lymphoma with early progression

**DOI:** 10.1186/s40364-020-00214-3

**Published:** 2020-08-26

**Authors:** Jialin Ma, Zheng Yan, Jiuyang Zhang, Wenping Zhou, Zhihua Yao, Haiying Wang, Junfeng Chu, Shuna Yao, Shuang Zhao, Peipei Zhang, Yuanlin Xu, Qingxin Xia, Jie Ma, Bing Wei, Shujun Yang, Kangdong Liu, Yongjun Guo, Yanyan Liu

**Affiliations:** 1 Department of Internal Medicine, Affiliated Cancer Hospital of Zhengzhou University & Henan Cancer Hospital, 127 Dong Ming Road, Zhengzhou, 450008 Henan China; 2 Department of Molecule and Pathology, Affiliated Cancer Hospital of Zhengzhou University & Henan Cancer Hospital, Zhengzhou, Henan China; 3 China-US (Henan) Hormel Cancer Institute, Zhengzhou, Henan China

**Keywords:** Diffuse large B-cell lymphoma, Early progression, CD79B, PIM1

## Abstract

**Background:**

Early progression after the first-line R-CHOP treatment leads to a very dismal outcome and necessitates alternative treatment for patients with diffuse large B-cell lymphoma (DLBCL). This study aimed to develop a genetic predictive model for early progression and evaluate its potential in advancing alternative treatment.

**Methods:**

Thirty-two hotspot driver genes were examined in 145 DLBCL patients and 5 DLBCL cell lines using next-generation sequencing. The association of clinical features, cell-of-origin, double expression, positive p53 protein, and gene alterations with early progression was analyzed, and the genetic predictive model was developed based on the related independent variables and assessed by the area under receiver operating characteristic. The potential of novel treatment based on the modeling was investigated in in-vitro DLBCL cell lines and in vivo xenograft mouse models.

**Results:**

The frequency of *CD79B* (42.86% vs 9.38%, *p* = 0.000) and *PIM1* mutations (38.78% vs 17.71%, *p* = 0.005) showed a significant increase in patients with early progression. *CD79B* and *PIM1* mutations were associated with complex genetic events, double expression, non-GCB subtype, advance stage and unfavorable prognosis. A powerful genetic predictive model (AUROC = 0.771, 95% CI: 0.689–0.853) incorporating lactate dehydrogenase levels (OR = 2.990, *p* = 0.018), *CD79B* mutations (OR = 5.970, *p* = 0.001), and *PIM1* mutations (OR = 3.021, *p* = 0.026) was created and verified in the other cohort. This modeling for early progression outperformed the prediction accuracy of conventional International Prognostic Index, and new molecular subtypes of MCD and Cluster 5. *CD79B* and *PIM1* mutations indicated a better response to inhibitors of BTK (ibrutinib) and pan-PIM kinase (AZD 1208) through repressing activated oncogenic signaling. Since the two inhibitors failed to decrease BCL2 level, BCL2 inhibitor (venetoclax) was added and demonstrated to enhance their apoptosis-inducing activity in mutant cells with double expression.

**Conclusions:**

The genetic predictive model provides a robust tool to identify early progression and determine precision treatment. These findings warrant the development of optimal alternative treatment in clinical trials.

## Background

Diffuse large B-cell lymphoma (DLBCL) is the most common subtype of non-Hodgkin’s lymphoma with great heterogeneity in genetics, manifestations, therapy responses, and prognoses. The first-line R-CHOP treatment has achieved a complete cure or yielded a long-term survival in over 60% of patients. However, the rest eventually succumbed to recurrent or refractory disease [1–4]. Particularly, those with early progression within less than 12 months (POD12) usually experienced a very dismal outcome and did not benefit much from salvage therapy in combination with autologous stem cell transplantation [5, 6]. Hence, it is necessary to provide them an alternative treatment beyond standard immunochemotherapy in the setting of frontline therapy. Firstly, it is important to introduce a powerful predictive model for POD12 in newly diagnosed DLBCL patients. Currently, International Prognostic Index (IPI) based on five clinical parameters and cell of origin (COO) classification into germinal-center B-cell-like (GCB), activated B-cell-like (ABC), and unclassified type 3 subtypes have been used for prognostic assessment in DLBCL [7, 8]. Concurrent BCL2 and c-MYC and/or BCL6 rearrangements, double expression (DE) of c-MYC and BCL2, and positive p53 protein expression have also been reported to be associated with poor outcomes [9–12]. The subset involving c-MYC and BCL2 and/or BCL6 rearrangements has been classified into high-grade double-hit or triple-hit B-cell lymphoma, for which intensive-dose regimens are recommended in the frontline therapy [11, 13]. However, accurate prediction of other prognostic models for POD12 remains elusive. In addition, optimal alternative treatment beyond standard immunochemotherapy has not been determined. It has been reported that the addition of novel targeted agents, such as bortizumab, lenalidomide, and ibrutinib, did not meet the primary endpoint of improving overall survival in clinical trials designed based on COO [14, 15].

Recently, many gene alterations have been identified using whole-exome and transcriptome sequencing in DLBCL samples [16–18]. Some of these alterations have been confirmed to drive tumor development and promote DLBCL cell proliferation and survival via regulating oncogenic signaling pathways. In this context, several genetic prognostic models incorporating gene alterations have been established to predict the outcomes, and outperformed the currently known models such as IPI, COO, and DE [17]. These prognostication tools have not only extended our understanding of DLBCL pathogenic mechanisms, but also uncovered potential opportunities for precision treatment strategies. For example, *CD79B* mutations involved in the B-cell receptor (BCR) signaling result in BCR-dependent activation of NF-κB [19]. Some new molecular subtypes are characteristic of *CD79B* mutations, such as MCD termed by both *CD79B* and *MYD88 L265P* mutations [18], and Cluster 5, which is a unique genetic signature of ABC DLBCL and enriched with *CD79B* mutations [16]. Ibrutinib, a BTK inhibitor, has been observed to induce better response in DLBCL patients with *CD79B* mutations [18, 20, 21]. Increasing evidence suggests that targeted agents should be evaluated in DLBCL clinical trials in the context of subtype-specific genetic aberrations and activating mutations that positively modulate oncogenic signaling pathways.

Herein, we analyzed a panel of 32 genes with high frequency of mutations in DLBCL, which have been reported to contribute to tumorigenesis and progression [18, 22]. A robust genetic predictive model for POD12 was established after evaluating the association of traditional prognostic factors and gene alterations with POD12. This genetic predictive model suggests a novel treatment strategy by targeting specific gene alterations, which was successfully confirmed using in in-vitro DLBCL cell lines and in-vivo xenograft mouse models.

## Methods

### Patients and cell lines

One hundred and forty-five patients with newly diagnosed DLBCL were enrolled in this study. The diagnosis of DLBCL was confirmed by at least two pathologists in accordance to the World Health Organization classification [23], and patients with double-hit and triple-hit were excluded from the study. All patients were treated with R-CHOP in the frontline setting. A cohort of 84 DLBCL patients was used for validation. Patient characteristics are shown in Supplementary Table 1. This study was reviewed and approved by the hospital Institutional Review Boards with informed consent of the patients.

Five human DLBCL cell lines were used in the study, including OCI-Ly8, Ros50, OCI-Ly3, OCI-Ly7, and Val. They have been authenticated and monitored for mycoplasma contamination.

### Next-generation sequencing

Genomic DNA was extracted from formalin-fixed paraffin-embedded tumor tissues of DLBCL patients using a QIAamp DNA FFPE Tissue kit (Qiagen) or from cultured cells using the TIANamp Genomic DNA kit (Tiangen). High-throughput DNA sequencing was performed on Illumina Genome Analyzer MIseq (Illumina) according to the manufacturer’s instructions. Quality of DNA libraries was assessed using a Bioanalyzer High Sensitivity DNA chip (Agilent Technologies). VarDict (v1.4.6) [24] and Varscan (v2.4.2) [25] were utilized to call single nucleotide polymorphism (SNP) and small indel from the BAM files. The variants were filtered including the aligned reads depth of variant over 500-fold with frequency of over 2%, and the allele frequency of lower than 5% in the 1000G, ESP or ExAC database.

### Immunohistochemistry

Immunohistochemistry was performed on 3-μm paraffin sections with an indirect immunoperoxidase method using antibodies against CD10 (Abcam, 1:500), BCL6 (Abcam, 1:500), MUM1 (Abcam 1:250), BCL2 (Abcam, 1:250), c-MYC (Abcam, 1:250), and p53 (Abcam, 1:50). COO subgroups were determined using the Han’s classification [26]. DE of BCL2 and c-MYC was defined as cut-off value of 50 and 40%, respectively. The cut-off value of 50% was considered as p53 protein positive.

### Cell proliferation

Cells in log growth phase were inoculated into 96-well plates in triplicate at a density of 2 × 10^5^/ml. After treatment with different concentrations of drugs, 20 μl of CCK-8 reagent (Meilunbio) was added, and continued to culture for another 4 h. Cell viability was quantified by reading absorbance at 450 nm on an automatic microplate reader (Thermo Fisher 1510 Vantaa, Finland).

### Cell apoptosis

After treatment with different drugs, 5 × 10^5^ cells were washed and resuspended in 100 μl of 1× binding buffer containing 5 μl Annexin-V (BD Pharmingen) and 5 μl 7-AAD (BD Pharmingen). Following incubation for another 15 min at room temperature in the dark, cell suspension was added with 400 μl of 1 × binding buffer, and then analyzed on a FACScan. The lower right-hand and the upper right-hand quadrant cells were considered apoptotic.

### Western blot

Total protein was extracted with RIPA buffer (Beyotime), and nuclear and cytoplasmic fractions were isolated using the Nuclear and Cytoplasmic Protein Extraction kit (Beyotime). Western blot was carried out following the standard protocol with the following primary antibodies, β-actin (Trans, 1:5000), BCL2 (Abcam, 1:1500), c-MYC (Abcam, 1:1500), phosphorylated-CDC25A (Abcam, 1:500), H_3_ (Abcam, 1:1000), MCL1 (CST, 1:1000), BCL-X_L_ (CST,1:1000), p65 (CST,1:1000), IκB-α (CST,1:1000). Protein bands were visualized using the enhanced chemiluminescence system (Beyotime) according to the manufacturer’s instruction.

### Xenograft mouse model

Six-week-old SCID mice (Charles River) were subcutaneously injected with OCI-Ly8 and Val cells in the posterior flank. When tumor sizes approached 150 mm^3^, mice were randomly divided into control and AZD1208 groups. AZD1208 (50 μg/g) was fed daily after being formulated in 0.5% CMC-Na solution. Tumor size was measured every other day and estimated by applying the following formula: (3.14 × length × width^2^)/6. Animals were maintained and manipulated in accordance with the principles of laboratory animal care under the Institutional Animal Care and Use Committee-approved protocol.

### Statistical analysis

Clinical features, molecular biomarkers, and genes mutations were compared using *t* -test for continuous variables and χ^2^- test for categorical variables. Predictive model was assessed using the area under receiver operating characteristic (AUROC). Progression-free survival (PFS) was calculated from the date of initial diagnosis to the time of recurrence, death or the last follow-up. Overall survival (OS) was measured from the date of initial diagnosis to the death or the last follow-up. PFS and OS were estimated using the Kaplan–Meier method and the log-rank test was used for comparison between groups. Statistical analysis was carried out using Statistical Package for the Social Sciences (SPSS) 21.0 software (SPSS Inc., Chicago, IL, USA). Statistical significance was defined as *p* < 0.05.

## Results

### *CD79B* and *PIM1* mutations are independently related to POD12 following R-CHOP treatment

**Fig. 1:**
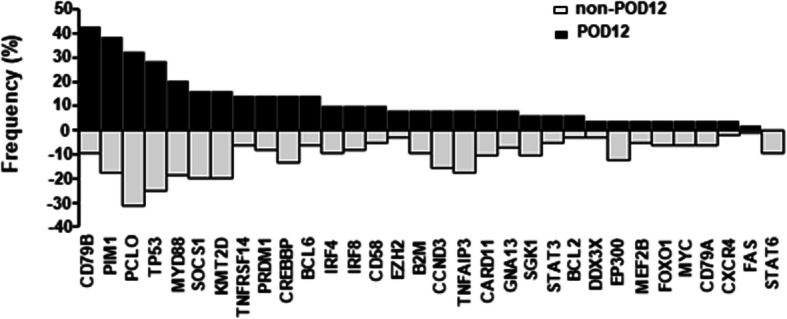
The diagram on frequencies of hotspot gene mutations

Total 32 hotspot driver genes were examined using next-generation sequencing in 145 newly diagnosed patients with DLBCL. All of these genes were mutated in 91.72% patients (133/145) with a median number of 4 (0–24), including single nucleotide variants, frameshift mutations, insertions, and deletions. SNPs were filtered according to the defined criteria. The frequency of *CD79B* (42.86% vs 9.38%, *p* = 0.000) and *PIM1* mutations (38.78% vs 17.71%, *p* = 0.005) showed a significant increase in patients with POD12 (*n* = 49) (Fig. 1), but no difference was found in median number of mutations (5 vs. 4, *p* = 0.287). The POD12 patients had very poor survival and almost all of them presented systemic relapse except for three patients involving central nervous system. When the associations of gender, age, ECOG score, Ann Arbor stage, LDH level, the number of extranodal involvement, IPI score, COO, DE, and positive p53 protein with POD12 were evaluated, univariate analysis displayed an obvious correlation with Ann Arbor stage (*p* = 0.019), LDH level (*p* = 0.001), IPI score (*p* = 0.014), and DE (*p* = 0.001) (Table 1). Multivariate analysis, including Ann Arbor stage, LDH level, DE, and gene mutations of *CD79B* and *PIM1*, revealed that LDH level (OR = 2.990, *p* = 0.018), *CD79B* (OR = 5.970, *p* = 0.001), and *PIM1* mutations (OR = 3.021, *p* = 0.026) were independently correlated with POD12.

**Table 1:** Clinical characteristics of patients grouped by POD12

Characteristics	POD12 (n = 49)	Non-POD12 (*n* = 96)	Univariate *p*-value	Multivariate *p*-value
Gender				
Male, n (%)	25 (51.0)	51 (53.1)	0.810	
IPI factors				
Age > 60 years, n (%)	17 (34.7)	23 (24.0)	0.171	
LDH level > normal, n (%)	32 (65.3)	35 (36.5)	**0.001**	**0.018**
Stage III or IV, n (%)	35 (71.4)	49 (51.0)	**0.019**	0.105
ECOG>1, n (%)	10 (20.4)	9 (9.4)	0.063	
Extranodal involvement > 1 site, n (%)	15 (30.6)	18 (18.8)	0.107	
IPI score				
Intermediate-high/high risk [3–5], n (%)	19 (38.8)	19 (19.8)	**0.014**	
Co-expression MYC and BCL2				
Yes, n (%)	18 (36.7)	13 (13.5)	**0.001**	0.096
COO				
Non-GCB, n (%)	22 (44.9)	46 (47.9)	0.901	
Positive p53 protein				
Yes, n (%)	14 (28.6)	18 (18.8)	0.127	
Gene mutations				
*PIM1*, n (%)	19 (38.8)	17 (17.7)	**0.005**	**0.026**
*CD79B*, n (%)	21 (42.9)	9 (9.4)	**0.000**	**0.001**

### *CD79B* and *PIM1* mutations are associated with complex genetic events and unfavorable prognosis

**Fig. 2:**
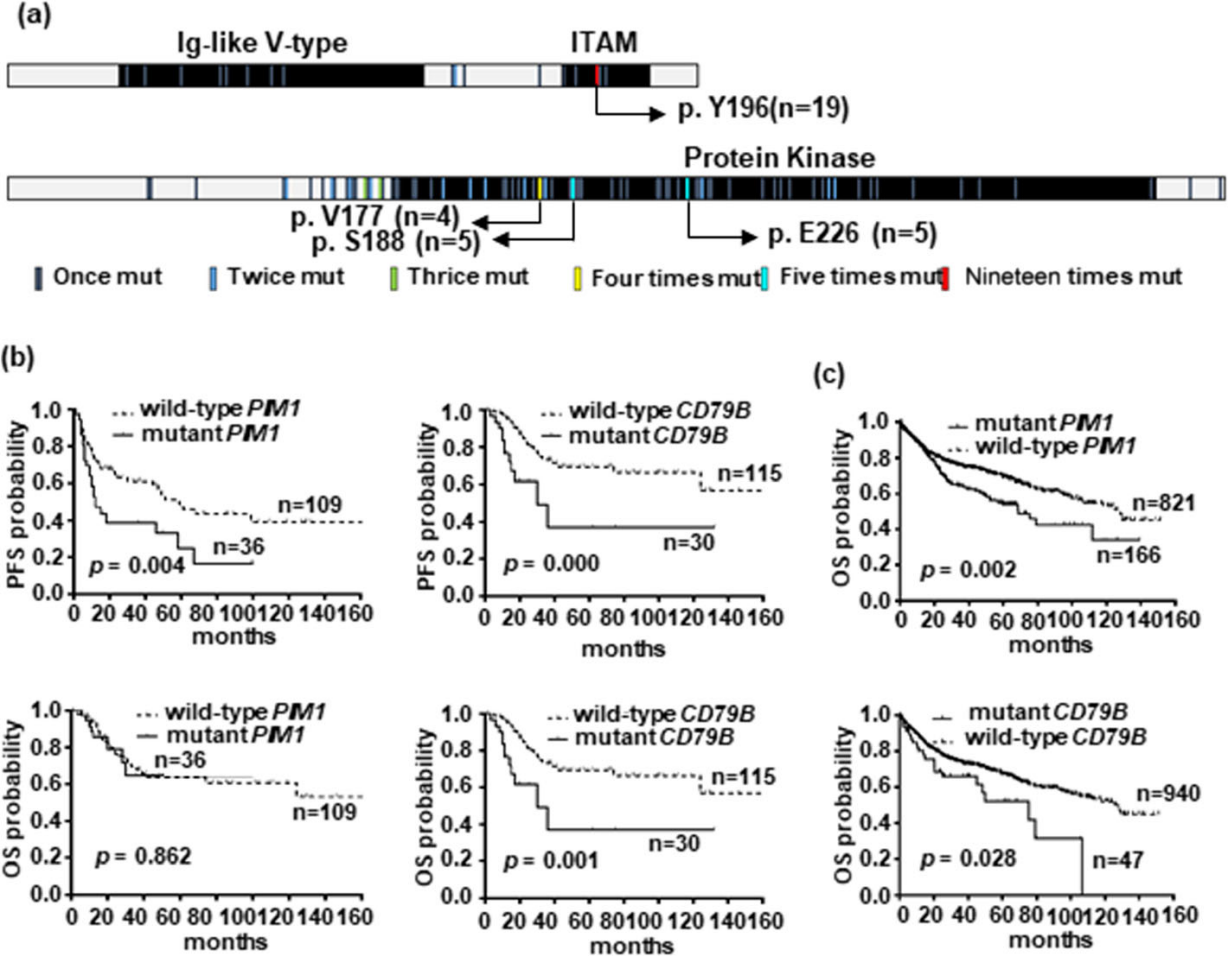
**The genetic features of** ***CD79B*** **and** ***PIM1*** **mutations and their association with survival.** (**a**) Complex genetic events were involved in the *CD79B* and *PIM1* mutations. (**b**) *PIM1*- (*n* = 36, *p* = 0.004) and *CD79B*-mutant (*n* = 30, *p* = 0.000) patients had poorer PFS than wild-type patients. *CD79B*-mutant patients displayed poorer OS (n = 30, *p* = 0.001), while those with *PIM1* mutation were indifferent (n = 36, *p* = 0.862). (**c**) *PIM1*- (*n* = 166, *p* = 0.002) and *CD79B*-mutant (*n* = 47, *p* = 0.028) patients were validated to have worse OS in a larger DLBCL cohort

Complex genetic events were seen involving in *CD79B* and *PIM1* mutations (Fig. 2a). There were 36 mutational sites occurring in 30 patients with *CD79B* mutations, including 8 in the Ig-like V type domain, 5 in the transmembrane domain, and 23 in the immunoreceptor tyrosine-based activation motif (ITAM). A prominent site was identified at the Y196 of ITAM in 63.3% (19/30) of patients. Moreover, 25 gene alterations were observed to accompany with *CD79B* mutations, only *PIM1* (14/36, *p* = 0.002) and *MYD88 L265P* (11/16, *p* = 0.000) mutations having significant correlation. 105 mutational sites of *PIM1* were seen in 36 patients, a majority of which occurred in the kinase domain with V177 (4/36, 11.1%), S188 (6/36, 16.7%) and E226 (6/36, 16.7%) having high frequency involvement. There were 30 gene alterations accompanying with *PIM1* aberrations, including *IRF4* (9/14, *p* = 0.001) and *MYD88 L265P* (10/16, *p* = 0.000) alterations having obvious correlation.

By analyzing clinicopathological features, *CD79B* mutations were significantly associated with DE (*p* = 0.001) and non-GCB subtype (*p* = 0.030), and *PIM1* mutations were statistically relevant to DE (*p* = 0.049) and advance stage (*p* = 0.023) (Supplementary Table 2). Patients with *CD79B* mutations manifested poorer PFS and OS than wild-type patients, while patients with *PIM1* mutations presented poorer PFS, but not OS (Fig. 2b). In a larger cohort [17], both *CD79B*- and *PIM1*-mutant patients were found to have worse survival than those with wild-type genes (Fig. 2c). These data indicate that *CD79B* and *PIM1* mutations are associated with complex genetic events and unfavorable prognosis.

### A robust predictive model for POD12 is created by incorporating the variables *CD79B* mutations, *PIM1* mutations, and LDH levels

**Fig. 3:**
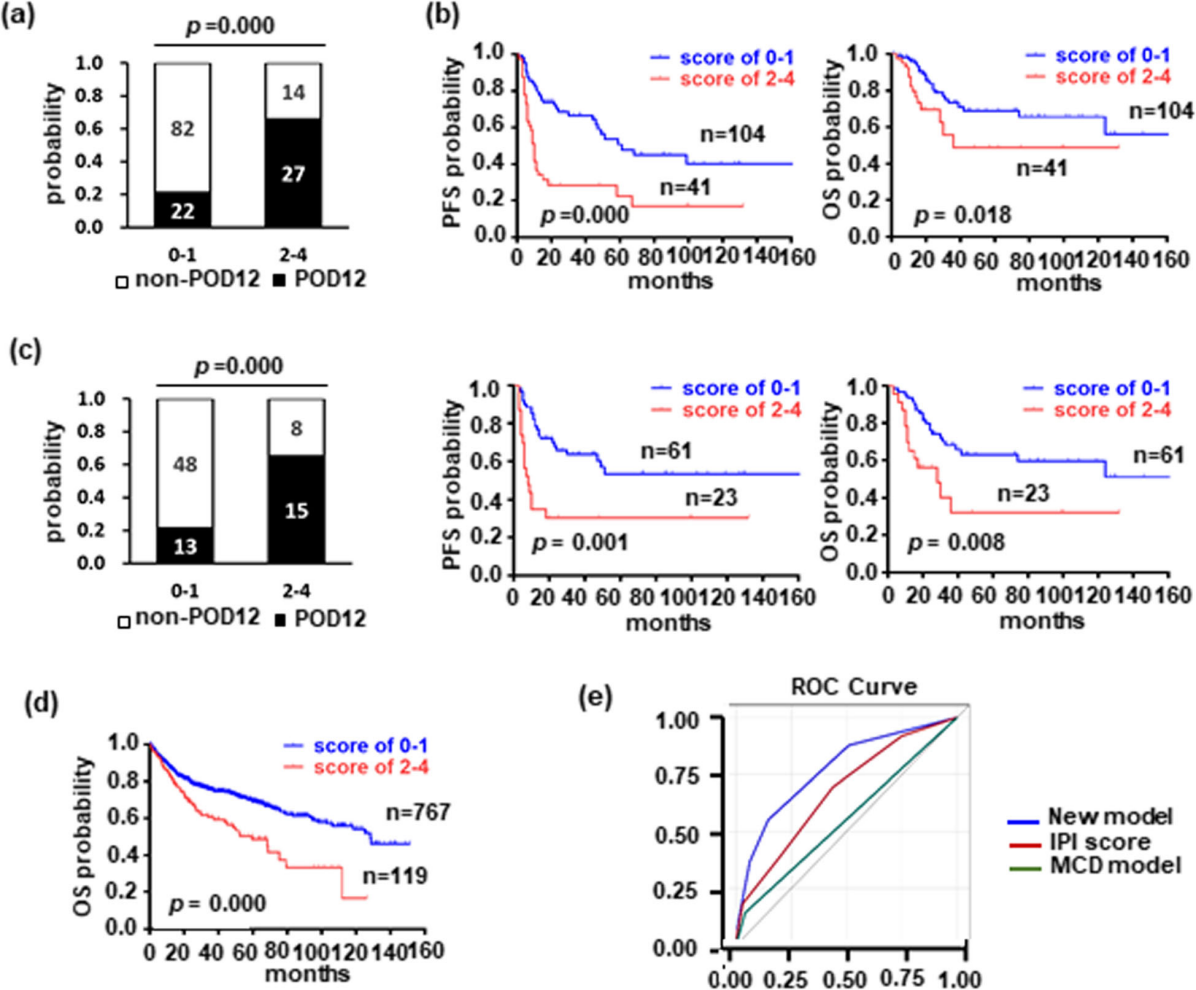
**The new genetic predictive model for POD12 including** ***CD79B*** **mutation,** ***PIM1*** **mutation, and LDH levels.** (a) The incidence of POD12 was significantly different between patients with scores of 0–1 (*n* = 104) and 2–4 (*n* = 41) based on the genetic predictive model (21.15% vs 65.85%, *p* = 0.000). (b) There was an inverse correlation on PFS (*p* = 0.000) and OS (*p* = 0.018) between patients with score of 0–1 (n = 104) and 2–4 (n = 41). (c) The genetic predictive model was validated in a cohort of 84 DLBCL cases. (d) The association of scores of 2–4 (*n* = 119) with poorer survival was confirmed in a larger cohort (*p* = 0.000). (e) The genetic predictive model for POD12 outperformed the IPI score and MCD subtype

We established a new genetic predictive model for POD12 after integrating LDH levels (OR = 2.990, *p* = 0.018), *CD79B* mutations (OR = 5.970, *p* = 0.001), and *PIM1* mutations (OR = 3.021, *p* = 0.026), which were independently related to POD12. In this genetic predictive model, LDH levels and *PIM1* mutations were defined as a score of 1, and *CD79B* mutations was assigned as a score of 2 based on their OR value. The analysis of AUROC (0.771, 95% CI: 0.689–0.853) demonstrated the model to have a good performance. With the highest Youden’s index of 0.4052, scores of 2–4 were recommended to distinguish low- and high-risk patients of POD12 with a sensitivity of 55.10% and a specificity of 85.42%. The incidence of POD12 was significantly increased in patients with scores of 2–4 compared with those with scoring 0–1 (21.15% vs 65.85%, *p* = 0.000), who also displayed poorer PFS and OS (Fig. 3a and b). The genetic predictive model was successfully validated in a cohort of 84 cases and in another larger cohort of 1001 patients [17] (Fig. 3c, and d). The cohort of 84 patients was enrolled in our center and exhibited similar features with the cohort of 145 cases. The larger cohort was derived from Reddy. B et al. study [17], which almost addressed these characteristics above mentioned except for positive p53 protein and special site of extranodal involvement. In comparison with the other two cohorts, it had higher proportion of the elderly (56.6% vs 27.6% vs 20.2%), IPI score 3–5 (44.3% vs 25.5% vs 23.8%), and lower frequency of *CD79B* mutation (4.7% vs 20.7% vs 20.2%) (Supplementary Table 1). The discrepancy may come from selection and/or ethnic deviation. However, patients with high-risk POD12 showed very poor survival in the larger cohort (*p* = 0.000) (Fig. 3d).

Our modeling was further compared with traditional IPI score and new molecular subtypes, MCD and Cluster 5, on the power for predicting POD12. The MCD subtype is defined as a co-occurrence of *CD79B* and *MYD88 L265P* mutations [18] and Cluster 5 is a genetic signature having frequent mutations in *CD79B*, *MYD88 L265P*, and *PIM1* [16]. The result showed that the value of this genetic model in predicting POD12 is superior to conventional IPI score and MCD subtype (Fig. 3e). The association of Cluster 5 with POD12 was not found (12/64, *p* = 0.576) by analyzing the data from Chapuy. B et al. study [16], either. Collectedly, we created a predictive model for POD12 with a powerful performance by incorporating the variables *CD79B* mutations, *PIM1* mutations, and LDH levels.

### *CD79B* and *PIM1* mutations indicate better response to BTK and pan-PIM kinase inhibitors, and BCL2 inhibitor enhances their apoptosis-inducing effects in cells with DE

**Fig. 4:**
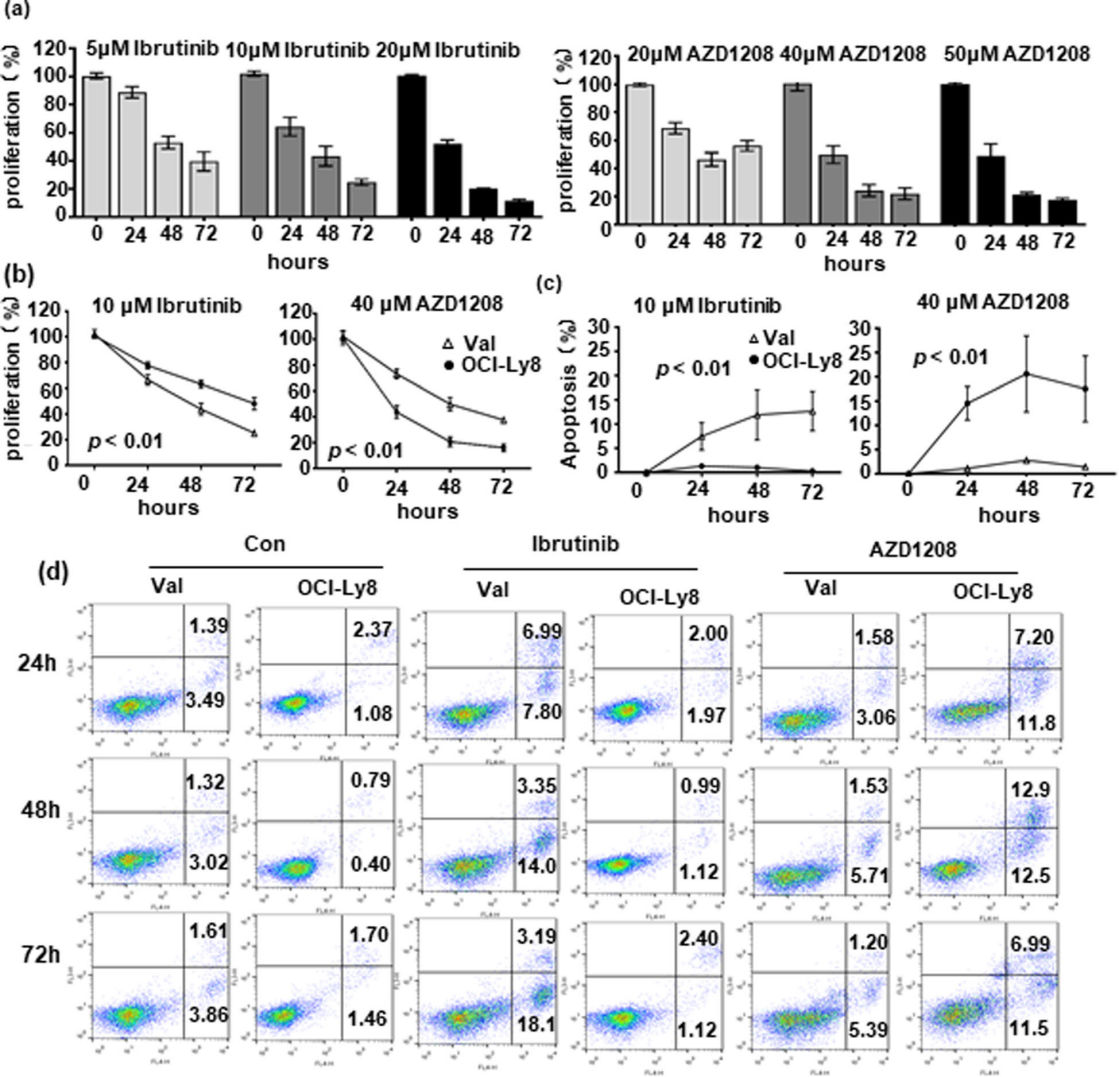
**Correlation of** ***CD79B*** **and** ***PIM1*** **mutations with BTK and pan-PIM inhibitors response.** (a) BTK inhibitor **(**Ibrutinib) and pan-PIM inhibitor (AZD 1208) showed a dose- and time-dependent growth inhibition in DLBCL cell lines. (b) *CD79B*-mutant Val cells and *PIM1*-mutant OCI-Ly8 cells were more susceptible to Ibrutinib (10 μM)- and AZD 1208 (40 μM)-induced growth inhibition (*p* < 0.01). (c) and (d) *CD79B*-mutant Val cells and *PIM1*-mutant OCI-Ly8 cells were more sensitive to Ibrutinib (10 μM)- and AZD 1208 (40 μM)-induced apoptosis (*p* < 0.01)

**Fig. 5:**
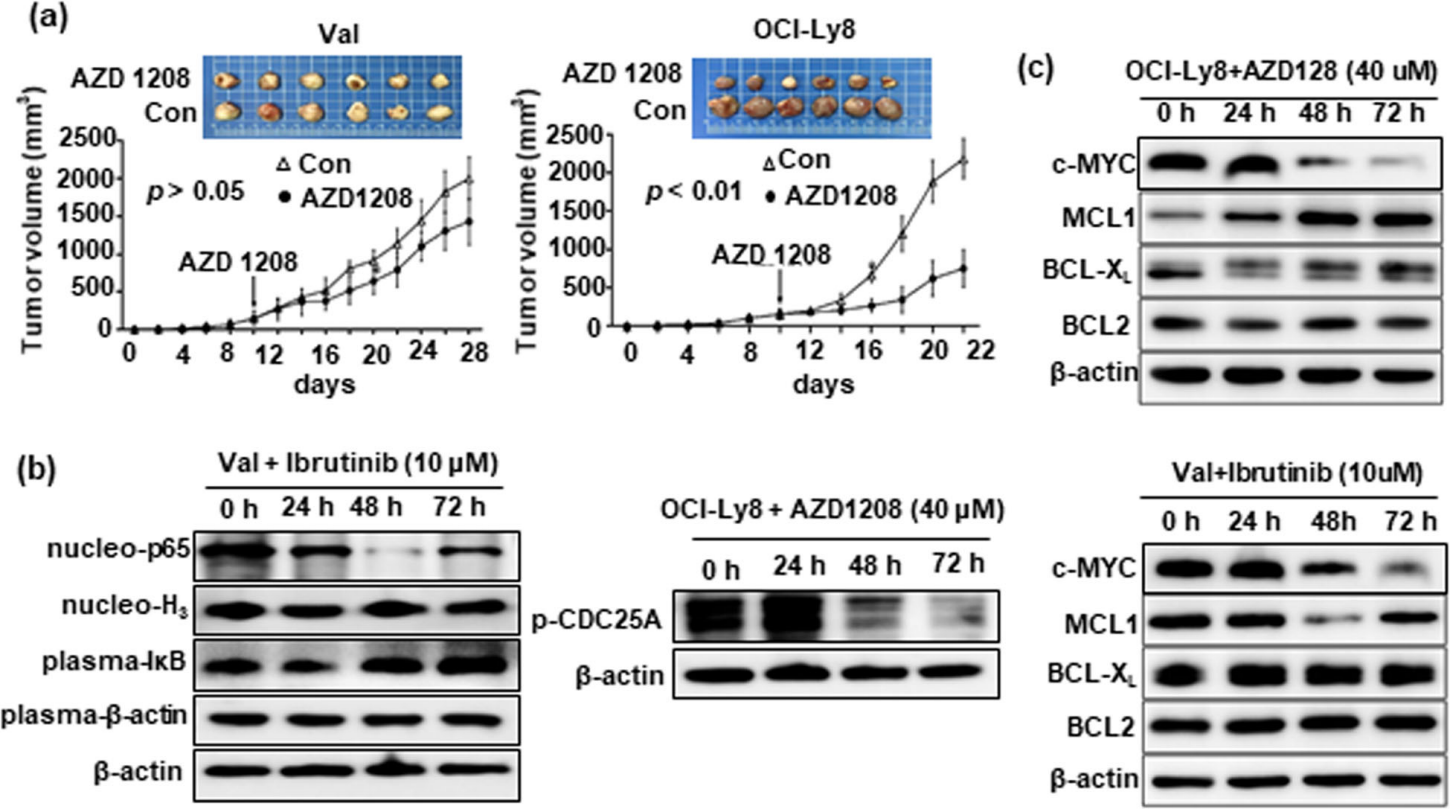
**Xenograft mouse models and mechanisms of BTK and pan-PIM Inhibitors.** (**a**) Tumor growth was significantly slowed down in *PIM1*-mutant OCI-Ly8 xenograft mice compared with *PIM1*-wildtype Val xenografts (*p* < 0.01). (**b**) Ibrutinib (10 μM) and AZD 1208 (40 μM) decreased the expression of key molecules in the related oncogenic pathways in *CD79B-* and *PIM1-*mutant cells. (**c**) Both Val and OCI-Ly8 cells expressed c-MYC and BCL2 proteins. Ibrutinib (10 μM) and AZD1208 (40 μM) induced the downregulation of c-MYC, but not BCL2 expression

A novel treatment was evaluated based on our predictive model. By sequencing a panel of 32 hotspot driver genes in 5 DLBCL cell lines, only Val cells had a *CD79B* mutation in the ITAM domain (T212M), and OCI-Ly8 cells had *PIM1* mutations in the kinase domain (S188N and L284F), which were both accompanied by mutations in MYC, BCL2, FOXO1, and CREBBP. Val and OCI-Ly8 cell lines were used to test the effects of *CD79B* and *PIM1* mutations on DLBCL cells sensitivity to BTK inhibitor ibrutinib and pan-PIM kinase inhibitor AZD 1208. Cell proliferation assay was carried out to determine experimental doses of ibrutinib and AZD1208 (Fig. 4a). We found that *CD79B*-mutant Val cells were more susceptible to 10 μM ibrutinib-induced growth inhibition and apoptosis when compared with *CD79B*-wildtype OCI-Ly8 cells (*p* < 0.01); *PIM1*-mutant OCI-Ly8 cells also presented a better response to 40 μM AZD 1208 than *PIM1*-wildtype Val cells (*p* < 0.01) (Fig. 4b, c, and d). The significance of *PIM1* mutations was further confirmed in xenograft mouse models. After AZD 1208 was given daily according to the protocol, tumor growth was significantly suppressed in OCI-Ly8 xenograft mice when compared with Val xenografts (Fig. 5a). These results suggest that *CD79B* and *PIM1* mutations make DLBCL cells sensitive to BTK and pan-PIM kinase inhibitors.

**Fig. 6:**
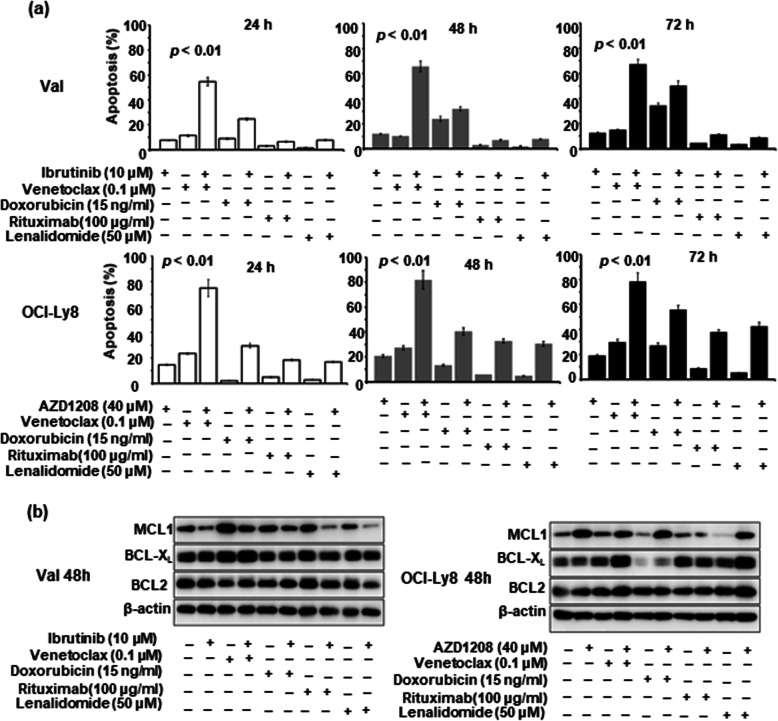
**The effect of other key drugs for DLBCL therapy on BTK and pan-PIM inhibitors.** (**a**) Venetoclax (0.1 μM) showed the most synergistic effect on Ibrutinib (10 μM)- and AZD 1208 (40 μM)-induced apoptosis (*p* < 0.01). (**b**) These drugs did not significantly decrease the BCL2 levels, although some of them exerted notable inhibitory on BCL-X_L_ and MCL1 levels

Next, we determined the action mechanisms of the two inhibitors by Western blot. A gradual increase of cytoplasmic IκB and a reduction of nuclear p65 were found after ibrutinib treatment for 24–72 h, while phosphorylated-CDC25A was decreased following AZD 1208 treatment for 24–72 h (Fig. 5b). Both c-MYC and BCL2 proteins were found to present in Val and OCI-Ly8 cells. However, c-MYC expression, but not BCL2 expression, was significantly reduced by ibrutinib and AZD1208 (Fig. 5c). Therefore, BCL2 inhibitor venetoclax was added to enhance the effectiveness of ibrutinib and AZD 1208. We picked 0.1 μM venetoclax for experiment based on the results of cell proliferation assay (Supplementary Fig. 1). Encouragingly, venetoclax did show a prominent synergistic effect when combined with ibrutinib and AZD 1208, even though it alone did not produce obvious apoptosis (Fig. 6a). The observed effect of venetoclax should be attributed to its inhibition of BCL2 function, since BCL2 levels were not affected (Fig. 6b). The combinatorial effect of other key agents for DLBCL therapy, including rituximab, doxorubicin, and lenalidomide, were also examined with ibrutinib and AZD 1208. Their doses in in-vitro assay were also chosen based on the results of cell proliferation assay (Supplementary Fig. 1). The results showed that 100 μg/ml rituximab, 15 ng/ml doxorubicin, and 50 μM lenalidomide only produced rather limited synergistic action of apoptosis with ibrutinib and AZD 1208 (Fig. 6a). Although some of them exerted inhibitory effects on BCL-X_L_ and MCL1, they have no effect on BCL2 expression (Fig. 6b), suggesting the importance of blocking BCL2 in promoting apoptosis of mutant cell with DE.

## Discussion

We observed a panel of 32 high-frequency mutated genes and a median number of 4 mutations in DLBCL patients, which are similar to the findings in the study containing larger amount of candidate cancer genes (CCGs) [16]. To make up for the deficiency of lacking patient-matched normal samples, we developed a computational method to filter germline variants and artifacts. Even though it did not completely exclude rare germline variants, some evidences have indicated that these rare germline variants have minimal effects on the detection of CCGs [16].

By excluding patients with double-hit and triple-hit from this study, we successfully identified a new subset of DLBCL patients prone to early progression and in need of frontline alternative treatment with the use of a novel genetic predictive model. Simultaneously, we demonstrated that our genetic predictive model for POD12 has the advantage over conventional prognostic models such as IPI, COO, DE, positive p53 protein, and new prognostic models like MCD subtype and Cluster 5 genetic signature. MCD reported by Schmitz et al. was a new subtype of DLBCL with *CD79B* and *MYD88 L265P* mutations and had inferior clinical outcomes following R-CHOP treatment [18]. Most of MCD subtypes could be ascribed to ABC DLBCL and had a tendency of extensive extranodal involvement [27]. Chapuy et al. integrated recurrent mutations, somatic copy number alterations, and structural variants to recognize a unique ABC-type Cluster 5, which exhibited frequent mutations in *CD79B* and *MYD88 L265P* and was associated with extranodal tropism and inferior survival [16]. *PIM1* mutations have been addressed to be frequent in patients with MCD subtype and cluster 5. However, their associations with POD12 have not been reported before.

Complex genetic events were observed in the *CD79B* and *PIM1* mutations, including their connectivity with *MYD88 L265P* and *IRF4*. The prominent mutation at Y196 of the CD79B ITAM domain has been demonstrated to enhance the BCR signaling [19]. Although the consequences of other CD79B mutations are not clear, current evidence supports their influence on the activation of the BCR signaling [28]. PIM1 has been linked to the initiation and progression of malignant phenotype by regulating cell cycle progression and inhibiting apoptosis. Many mutational sites of *PIM1* were observed with predominant mutations at V177, S188 and E226 of the kinase domain. *PIM1* alterations have been demonstrated to affect the structural stability and kinase activity of PIM1 [29, 30]. It was confirmed about unfavorable features of advanced stage, non-GCB, and DE and poor survival in *CD79B-* and *PIM1-*mutant patients.

In agreement with the previous study [31], we demonstrated that *CD79B* and *PIM1* mutations signal better response to BTK and pan-PIM kinase inhibitors by suppressing oncogenic signaling. However, these two inhibitors induced limited apoptosis in *CD79B*- or *PIM1*-mutant cells with DE. Later mechanistic studies revealed that they decreased the expression of c-MYC, but had no impacts on BCL2, who persistent expression largely reduced the drug potency. Indeed, DLBCL patients responding to ibrutinib often experience a rapid PD in clinical practice [20]. A strong synergy of venetoclax with BTK and pan-PIM kinase inhibitors was found in these DE cells, although it alone did not induced obvious apoptosis. Some studies have reported that venetoclax alone rarely exhibits a notable efficacy in other B-cell malignancies except chronic lymphocytic leukaemia [32, 33]. We also found that other key agents for DLBCL therapy, such as rituximab, doxorubicin, and lenalidomide, produced less synergistic activity to BTK and PIM1 inhibitors-induced apoptosis. Even though some of them reduced the expression of BCL-X_L_ and MCL1, they did not significantly affect the BCL2 expression. These findings suggest a key role of blocking BCL2 in promoting apoptosis of DLBCL cells with DE.

## Conclusions

In summary, we established a novel genetic predictive model for POD12, which powerfully identified a new subset of DLBCL patients prone to early progression and in need of alternative treatment beyond standard immunochemotherapy in the frontline setting. The genetic predictive model suggests precision therapy by targeting special oncogenic signaling and anti-apoptotic proteins in these high-risk patients for POD12.

## Supplementary information

**Additional file 1: Table S1.** The patient characteristics of three cohorts

**Additional file 2: Table S2.** Clinical characteristics of patients with *PIM1* or *CD79B* mutations

**Additional file 3: Fig. S1.** The inhibitory doses of venetoclax, rituximab, doxorubicin, and lenalidomide in in-vitro cell proliferation assay.

## Data Availability

Not applicable.

## References

[1] Ngo L (2008). Prognostic factors in patients with diffuse large B cell lymphoma: before and after the introduction of rituximab. Leuk Lymphoma.

[2] Coiffier B (2010). Long-term outcome of patients in the LNH-98.5 trial, the first randomized study comparing rituximab-CHOP to standard CHOP chemotherapy in DLBCL patients: a study by the Groupe d'Etudes des Lymphomes de l'Adulte. Blood.

[3] Costa LJ (2017). Diffuse large B-cell lymphoma with primary treatment failure: ultra-high risk features and benchmarking for experimental therapies. Am J Hematol.

[4] Crump M (2017). Outcomes in refractory diffuse large B-cell lymphoma: results from the international SCHOLAR-1 study. Blood.

[5] Gisselbrecht C (2010). Salvage regimens with autologous transplantation for relapsed large B-cell lymphoma in the rituximab era. J Clin Oncol.

[6] Epperla N (2019). Postrelapse survival in diffuse large B-cell lymphoma after therapy failure following autologous transplantation. Blood Adv.

[7] International Non-Hodgkin's Lymphoma Prognostic Factors, P., *A predictive model for aggressive non-Hodgkin's lymphoma.* N Engl J Med, 1993. **329**(14): p. 987–94. 10.1056/NEJM199309303291402 8141877

[8] Alizadeh AA (2000). Distinct types of diffuse large B-cell lymphoma identified by gene expression profiling. Nature.

[9] Horn H (2013). MYC status in concert with BCL2 and BCL6 expression predicts outcome in diffuse large B-cell lymphoma. Blood.

[10] Johnson NA (2012). Concurrent expression of MYC and BCL2 in diffuse large B-cell lymphoma treated with rituximab plus cyclophosphamide, doxorubicin, vincristine, and prednisone. J Clin Oncol.

[11] Cheah CY (2015). A clinician's guide to double hit lymphomas. Br J Haematol.

[12] Xu-Monette ZY (2012). Mutational profile and prognostic significance of TP53 in diffuse large B-cell lymphoma patients treated with R-CHOP: report from an international DLBCL rituximab-CHOP consortium program study. Blood.

[13] Aukema SM (2011). Double-hit B-cell lymphomas. Blood.

[14] Davies A (2019). Gene-expression profiling of bortezomib added to standard chemoimmunotherapy for diffuse large B-cell lymphoma (REMoDL-B): an open-label, randomised, phase 3 trial. The Lancet Oncology.

[15] Younes A (2019). Randomized phase III trial of Ibrutinib and rituximab plus cyclophosphamide, doxorubicin, vincristine, and prednisone in non-germinal center B-cell diffuse large B-cell lymphoma. J Clin Oncol.

[16] Chapuy B (2018). Molecular subtypes of diffuse large B cell lymphoma are associated with distinct pathogenic mechanisms and outcomes. Nat Med.

[17] Reddy A (2017). Genetic and functional drivers of diffuse large B cell lymphoma. Cell.

[18] Schmitz R (2018). Genetics and pathogenesis of diffuse large B-cell lymphoma. N Engl J Med.

[19] Davis RE (2010). Chronic active B-cell-receptor signalling in diffuse large B-cell lymphoma. Nature.

[20] Wilson WH (2015). Targeting B cell receptor signaling with ibrutinib in diffuse large B cell lymphoma. Nat Med.

[21] Phelan JD (2018). A multiprotein supercomplex controlling oncogenic signalling in lymphoma. Nature.

[22] Miao Y (2019). Genetic alterations and their clinical implications in DLBCL. Nat Rev Clin Oncol.

[23] Sabattini E (2010). WHO classification of tumours of haematopoietic and lymphoid tissues in 2008: an overview. Pathologica.

[24] Lai Z (2016). VarDict: a novel and versatile variant caller for next-generation sequencing in cancer research. Nucleic Acids Res.

[25] Koboldt DC (2012). VarScan 2: somatic mutation and copy number alteration discovery in cancer by exome sequencing. Genome Res.

[26] Hans CP (2004). Confirmation of the molecular classification of diffuse large B-cell lymphoma by immunohistochemistry using a tissue microarray. Blood.

[27] Chapuy B (2016). Targetable genetic features of primary testicular and primary central nervous system lymphomas. Blood.

[28] Kraus M (2004). Survival of resting mature B lymphocytes depends on BCR signaling via the Igalpha/beta heterodimer. Cell.

[29] Lori C (2013). Effect of single amino acid substitution observed in cancer on Pim-1 kinase thermodynamic stability and structure. PLoS One.

[30] Kumar A (2005). Crystal structures of proto-oncogene kinase Pim1: a target of aberrant somatic hypermutations in diffuse large cell lymphoma. J Mol Biol.

[31] Kuo HP (2016). The role of PIM1 in the ibrutinib-resistant ABC subtype of diffuse large B-cell lymphoma. Am J Cancer Res.

[32] Roberts AW (2016). Targeting BCL2 with Venetoclax in relapsed chronic lymphocytic leukemia. N Engl J Med.

[33] Thijssen R, Roberts AW (2019). Venetoclax in lymphoid malignancies: new insights, More to Learn. Cancer Cell.

