# Supplementary material for: A genetic predictive model for precision treatment of diffuse large B-cell lymphoma with early progression
Source: Biomark Res. 2020 Aug 26;8:33. doi: 10.1186/s40364-020-00214-3 (PMC7448459; doi:10.1186/s40364-020-00214-3)
Supplement: Supplementary file 1 — Additional file 1: Table S1. The patient characteristics of three cohorts [file 40364_2020_214_MOESM1_ESM.docx]

**Supplementary Table 1: The patient characteristics of three cohorts**

| Characteristics | Cohort 1 (n=145) | Cohort 2 (n=84) | Cohort 3 (n=1001) |
| --- | --- | --- | --- |
| Gender |  |  |  |
| Male, n (%) | 76（52.4） | 42（50.0） | 564（56.5） |
| IPI factors |  |  |  |
| Age >60 years, n (%) | 40（27.6） | 17（20.2） | 538（56.6） |
| Serum LDH >normal, n (%) | 67（46.2） | 39（46.4） | 493（55.3） |
| Stage III or IV, n (%) | 84（57.9） | 52（61.9） | 590（60.6） |
| ECOG PS ＞1, n (%) | 19（13.1） | 13（15.5） | 242（26.4） |
| Extranodal involvement >1 site, n (%) | 33（22.8） | 16（19.0） | 211（23.1） |
| IPI score |  |  |  |
| Intermediate-high and high risk (3-5), n (%) | 37（25.5） | 20（23.8） | 337（44.3） |
| Co-expression MYC and BCL2 |  |  |  |
| Yes, n (%) | 31（21.4） | 11（13.1） | 154（19.9） |
| COO |  |  |  |
| Non-GCB, n (%) | 68（46.9） | 45（53.6） | 416（53.6） |
| Positive p53 protein |  |  |  |
| Yes, n (%) | 32（22.1） | 13（15.5） | NA |
| Gene mutation |  |  |  |
| *PIM1*, n (%) | 36（24.8） | 16（19.0） | 166（16.6） |
| *CD79B*, n (%) | 30（20.7） | 17（20.2） | 47（4.7） |
| Cycle number of R-CHOP |  |  |  |
| 4 | 56（38.6） | 36（42.9） | NA |
| 5 | 39（26.9） | 23（27.4） | NA |
| 6 | 50（34.5） | 25（29.8） | NA |
| CNS IPI score |  |  |  |
| Low risk（0-1） | 69（47.6） | 39（46.4） | NA |
| Intermediate risk（2-3） | 62（42.8） | 39（46.4） | NA |
| High risk（4-6） | 14（9.6） | 6（7.2） | NA |
