# Supplementary material for: A genetic predictive model for precision treatment of diffuse large B-cell lymphoma with early progression
Source: Biomark Res. 2020 Aug 26;8:33. doi: 10.1186/s40364-020-00214-3 (PMC7448459; doi:10.1186/s40364-020-00214-3)
Supplement: Supplementary file 2 — Additional file 2: Table S2. Clinical characteristics of patients with PIM1 or CD79B mutations [file 40364_2020_214_MOESM2_ESM.docx]

| Supplementary Table 2: Clinical characteristics of patients with *PIM1* or *CD79B* mutations | | | | | | |
| --- | --- | --- | --- | --- | --- | --- |
| Characteristics | ***PIM1*** | | ***p*-value** | ***CD79B*** | | ***p*-value** |
|  | **Mutant**  **(n=36)** | **Wild-type**  **(n=109)** |  | **Mutant**  **(n=30)** | **Wild-type**  **(n=115)** |  |
| IPI factors |  |  |  |  |  |  |
| Age >60 years, n (%) | 10(27.8) | 30(27.5) | 0.976 | 11(36.7) | 29(25.2) | 0.211 |
| LDH elevated, n (%) | 18(47.2) | 50(45.9) | 0.667 | 16(53.3) | 51(44.3) | 0.379 |
| Stages III-IV, n (%) | 15(41.7) | 69(63.3) | **0.023** | 17(56.7) | 67(58.3) | 0.875 |
| ECOG ≥2, n (%) | 6(16.7) | 13(11.9) | 0.656 | 4(13.3) | 15(13.0) | 1.000 |
| Extranodal sites >1, n (%) | 8(22.2) | 25(22.9) | 0.929 | 7(23.3) | 26(22.6) | 0.933 |
| IPI score |  |  |  |  |  |  |
| Intermediate-high / high risk (3-5), n (%) | 8(22.2) | 30(27.5) | 0.531 | 10(33.3) | 28(24.3) | 0.319 |
| Co-express MYC and BCL2 |  |  |  |  |  |  |
| Yes, n (%) | 12(33.3) | 19(17.4) | **0.049** | 12(40.0) | 19(16.5) | **0.001** |
| COO |  |  |  |  |  |  |
| Non-GCB, n (%) | 22(61.1) | 46(42.2) | 0.060 | 19(63.3) | 49(42.6) | **0.030** |
| Positive P53 expression |  |  |  |  |  |  |
| Yes, n (%) | 9(25.0) | 23(21.1) | 0.623 | 6(20.0) | 26(22.6) | 0.948 |
| Extranodal involvement |  |  |  |  |  |  |
| Yes, n (%) | 22(61.1) | 70(64.2) | 0.737 | 19(63.3) | 73(63.5) | 0.988 |
| Accompanying mutation |  |  |  |  |  |  |
| *IRF4*, n (%) | 9(25.0) | 5(4.6) | **0.001** |  |  |  |
| *CD79B*, n (%) | 14(38.9) | 16(14.7) | **0.002** |  |  |  |
| *MYD88* L265P, n (%) | 10(27.8) | 6(5.5) | **0.000** | 11(36.7) | 5(4.3) | **0.000** |
