# Supplementary figures and images for: A genetic predictive model for precision treatment of diffuse large B-cell lymphoma with early progression
Source: Biomark Res. 2020 Aug 26;8:33. doi: 10.1186/s40364-020-00214-3 (PMC7448459; doi:10.1186/s40364-020-00214-3)

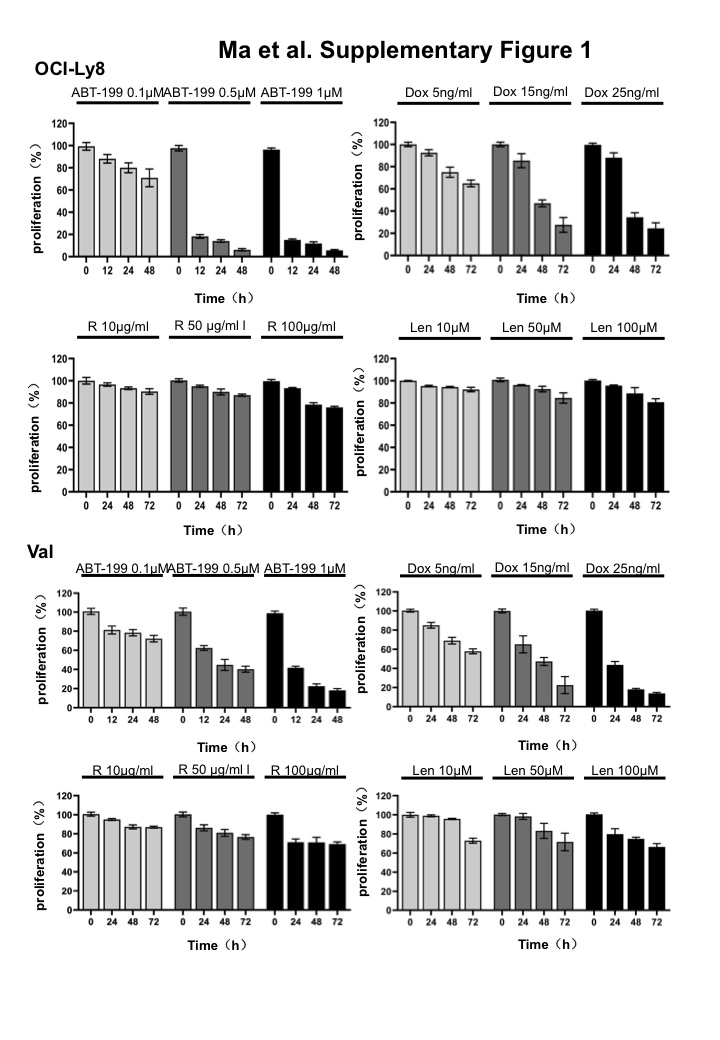

Supplement: Supplementary file 3 — Additional file 3: Fig. S1. The inhibitory doses of venetoclax, rituximab, doxorubicin, and lenalidomide in in-vitro cell proliferation assay. [file 40364_2020_214_MOESM3_ESM.jpg]
